# Porosity analysis of MTA and Biodentine cements for use in endodontics by using micro–computed tomography

**DOI:** 10.4317/jced.54688

**Published:** 2018-03-01

**Authors:** Fabricio Guerrero, Esther Berástegui

**Affiliations:** 1 DDS, MS, PhD Student, Department of Odonto-Stomatology, Faculty of Dentistry, University of Barcelona; 2 MD, DDS, PhD, Professor of Endodontic, Department of Odonto-Stomatology, Faculty of Dentistry, University of Barcelona

## Abstract

**Background:**

The purpose of this study is to compare the porosity of two repair cements, White ProRoot® MTA and Biodentine®. These samples were analyzed by using micro-computed microtomography.

**Material and Methods:**

Sixteen samples were used in the study that were divided according to the composition of the materials used. White ProRoot® MTA (n = 8) and Biodentine® (n = 8) were the samples prepared according to the manufacturer’s instructions. They were placed in silicone molds of 5 ± 0.1mm in height and an internal diameter of 5 ± 0.1mm, 24 hours after its preparation, the samples were scanned through a micro-CT, the porosity results were analyzed statistically by independent “t” tests.

**Results:**

It is evident that Biodentine® has better porosity properties than ProRoot® MTA. The results of the study quantify a smaller number of pores per surface, a smaller volume in each pore per mm3 and a lower total porosity present in samples of Biodentine® unlike ProRoot® MTA samples which is larger in both.

**Conclusions:**

The results obtained in computerized microtomography endodontic biomaterial samples concluded that Biodentine® has a lower porosity than ProRoot® MTA.

** Key words:**Porosity, microleakage, micro-CT, endodontic cements.

## Introduction

When performing endodontic treatment, for the sealing technique to be successful generally a sealing material and a base material are required, which are generally gutta-percha cones (1-3). Currently, there is a great diversity of endodontic materials classified according to their composition and their physicochemical properties (4-6).

There are studies that have analyzed that the solubility of a duct sealing material is in permanent relationship with the porosity that this material may have (7,8). The porosity and other defects of the microstructure of the endodontic sealant can produce foci of structural weakness and tensile strength of the material, producing microcracks (9).

Microcracks are defined as a decrease in the partial or total strength of a sealant, which can cause leakage within the endodontic cement in the root canal (10). Therefore, when we use the term pores when we speak of an endodontic material, we refer to the interaction between its physical properties and the type of mixture that was used to produce the material. Sealing or duct repair materials that are mixed manually according to the studies that have been made are more prone to subjective factors induced by the operator, thus producing more structural defects (11,12).

When we talk about repairing cements, we can say that there is a wide range of endodontic materials that are classified according to their composition. The most commonly used restorative materials in endodontics in both *in vitro* and *in vivo* studies are cements based on mineral trioxide aggregate (MTA) such as ProRoot® MTA (13) and are based on calcium silicates found in Biodentine® (14,15).

Micro-computed tomography (micro-CT) is being used as a tool for the quantitative and qualitative analysis of endodontic materials, and due to its quality of not manipulating the sample the technique used in the current studies of porosity analysis (4,16-19). In addition, the images obtained in the micro-CT allow a subsequent analysis of the material, resulting in a 3D scan of the analyzed material, which will allow observance of its internal structure and the ability to analyze the porosity of the material (18). Currently there is no study of the porosity between ProRoot® MTA (Dentsply Maillefer, Ballaigues, Switzerland) and Biodentine® (Septodont, Saint Maur des Fossés, France) that has been analyzed by micro-CT.

The objective of this *in vitro* study is to perform this comparison of the porosity between these two endodontic repair cements.

## Material and Methods

In this *in vitro* study, silicone tubes were used as molds to place the repair materials (n = 16), each silicone mold had a height of 5 ± 0.1mm and an internal diameter of 5 ± 0.1mm, filled with White ProRoot® MTA (n = 8) and the other repair cement used was Biodentine® (n = 8). All samples were prepared by a single operator following the manufacturer’s instructions for powder-to-liquid ratios, preparation time and setting time.

-Sample Preparation

The silicone molds cleaned their interior light and were placed on a microscope slide. Subsequently, a repair cement was mixed, until the eight samples per group were made according to the material used, in which the endodontic cement was placed by means of an amalgam holder and a metal spatula to fill the silicone molds and in the same way with all the samples of the study. We waited 24 hours in which the materials merged completely according to the indications of the manufacturers before passing to the analysis by means of the micro-CT (SkyScan 1174, Bruker micro-CT, Kontich, Belgium).

The silicone mold was not removed from repairing cements because, being a material that allows the passage of X-rays that produce the micro-CT does not influence or alter the study. In each sample that was found to be defective due to voids in the repair material in the silicone mold, the sample showing the defect was removed and replaced with a new sample.

-Scanning of micro-CT

The samples were scanned using a micro-CT (SkyScan 1174, Bruker micro-CT, Kontich, Belgium). The following scan parameters were applied: 50 kV and 800 μA voltage source, 9.6 μm pixel size, 0.80 ° rotation to achieve 180 ° total rotation and exposure time of 16000 ms. Using NRecon software (Skyscan), the 345 images obtained from the scan were reconstructed to show two-dimensional slices of the internal structure of the ProRoot® MTA and Biodentine® samples. Three-dimensional reconstruction, volumetric analysis and measurement of the pore volume were analyzed using CTan and CTVol (SkyScan) software of 450 cross sections of each sample.

-Statistical Analysis

The porosity values present in the endodontic repair cement samples analyzed in the study were compared using the student’s “t” tests for independent samples (SPSS 24.0, SPSS Inc., Chicago, IL). A value of *p* <0.05 was considered statistically significant.

## Results

**Figure 1:**
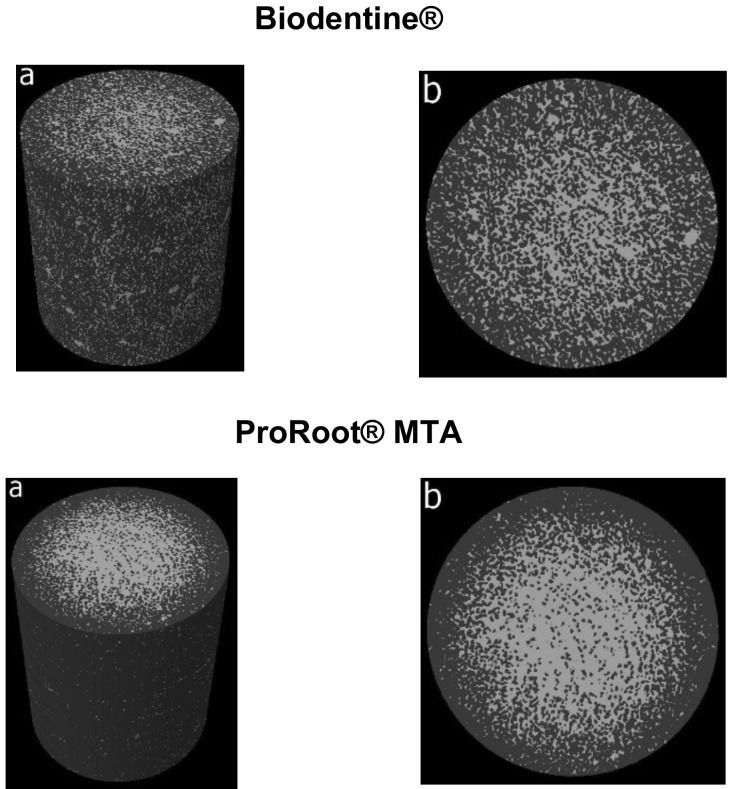
Porosity images of Biodentine® and ProRoot® MTA obtained through micro-CT. (a) 3D model with Presence of endodontic material and porosity. (b) Cross section of the repair material and present porosity of Biodentine® and ProRoot® MTA. The white color represents the presence of pores and the gray is the repair material without porosity.

Through the sagittal and transverse sections of the samples (Fig. 1), it was possible to quantify the volume of each pore per mm3, the number of pores per surface and the total porosity present in the endodontic materials, both based on MTA and based on calcium silicate ( Table 1).

**Table 1:**
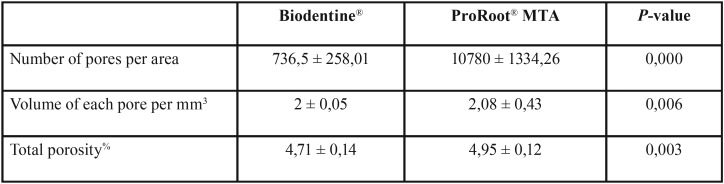
Number of pores per area, volume of each porosity per mm3 and total porosity of Biodentine® and ProRoot®.

It has been found that the repair material based on calcium silicate Biodentine®, has better properties in regard to porosity unlike the material based on mineral trioxide aggregate ProRoot® MTA, since the results of Biodentine® in what refers to the amount of pores per surface is notably lower than those presented by MTA (*P* = 0.000). In addition, the volume of each pore per mm3 in Biodentine® is smaller in volume than the MTA (*P* = 0.003) and the total porosity is also smaller in Biodentine® (*P* = 0.006).

## Discussion

Various techniques have been used for the assessment of porosity in endodontic materials, including dye staining (20), protein loss (21), bacteria leakage (22), mercury porometry (23), capillary flow porometry (24) and scanning electron microscopy (25). Computed microtomography is a three-dimensional imaging technique that leaves the sample intact and is being used as an alternative means to determine the porosity of a biomaterial (19).

The use of computed microtomography in endodontics has been one of the specialties in which the micro-CT is being used, since the endodontic materials can be evaluated as well as the techniques of duct filling (26,27).

Behr et al. (28) reported that the powder-liquid relationship, temperature and porosity can change the mechanical properties of endodontic cements. Therefore, the variables related to the mixing and placement of the material are key factors that influence the performance of endodontic cements. It reaffirms Ørstavik *et al.* (29) since it refers that the physical properties of root canal sealant and repair materials vary according to the composition and handling of each material.

Mutal (30) analyzed the repercussions of coronary patency on endodontically treated teeth in their study, in which they stated that even if a good obturation treatment is performed, its durability depends on several factors, including coronal filling and the use of a cement sealer that does not produce porosity, because the passage of bacteria is not only of coronal origin but also of apical origin. Mutal & Gani (8) corroborated it in a later study, in which they concluded that if a sealant has a high porosity it shows a high microfiltration, thus allowing the periradicular tissue fluids to penetrate the root canal system.

In an *in vitro* study carried out by Basturk *et al.* (19), they analyzed various properties of the MTA-based endodontic materials, including porosity, comparing two ProRoot® MTA and MTA Angelus® materials. These samples were analyzed by means of a micro-CT and the results concluded that there is no difference between these two repair cements as regards the porosity of the material. In the same way De Souza *et al.* (16) conducted an *in vitro* study in which they compared the porosity of four restorative endodontic materials, Ceramicrete®, iRoot BP Plus®, ProRoot® MTA and Biodentine®. These materials were analyzed and evaluated by a micro-CT to calculate the porosity, and the results affirmed that there is no difference in porosity between these materials. However, Mokeem *et al.* (4) stated that the resin-based materials produce less porosity inside the material, since they conducted a study comparing RealSeal®, EndoRez®, GuttaFlow and TubliSeal®, through a micro-CT. The results and conclusions of the aforementioned studies differ from those obtained in the present study since at the time of comparing ProRoot® MTA with Biodenitne® to evaluate the repair material that produces less porosity, the results showed that both materials have porosity, but that Biodentine® is the repair cement that has the least microfiltration inside the material and the results have a statistically significant difference.

## Conclusions

Within the limitations presented by the *in vitro* study, the results of the analysis of the images obtained through the micro-CT in the samples of the repairing cements with regard to porosity, it is observed that the repair cement Biodentine® presents less porosity compared to the porosity present in ProRoot® MTA.
